# Race/Ethnicity Among Children With COVID-19–Associated Multisystem Inflammatory Syndrome

**DOI:** 10.1001/jamanetworkopen.2020.30280

**Published:** 2020-11-30

**Authors:** Ellen H. Lee, Kelsey L. Kepler, Anita Geevarughese, Rachel Paneth-Pollak, Marie S. Dorsinville, Stephanie Ngai, Kathleen H. Reilly

**Affiliations:** 1 Incident Command System Surveillance and Epidemiology Section, New York City Department of Health and Mental Hygiene, Long Island City, New York

## Abstract

This cohort study describes the distribution of race/ethnicity among cases of coronavirus disease 2019 (COVID-19)–associated multisystem inflammatory syndrome in children reported to the New York City Department of Health and Mental Hygiene.

## Introduction

Reports^1,2,3^ suggest that a high proportion of cases of coronavirus disease 2019 (COVID-19)–associated multisystem inflammatory syndrome in children (MIS-C) occur among Black and Hispanic children. However, those published reports lack population-level data to contextualize the racial/ethnic distribution of cases. Here, we describe the distribution of race/ethnicity among MIS-C cases reported to the New York City (NYC) Department of Health and Mental Hygiene (DOHMH) and examine incidence rates by race/ethnicity to quantify the burden of MIS-C compared with COVID-19 hospitalizations.

## Methods

This cohort study involved data collected through routine public health surveillance; it was determined to be minimal risk and exempt by the DOHMH institutional review board. Consent was not required for the use of deidentified data. This study follows the Strengthening the Reporting of Observational Studies in Epidemiology (STROBE) reporting guideline.

On May 4, 2020, DOHMH required reporting of individuals younger than 21 years hospitalized in NYC with findings suggestive of MIS-C.^4^ Medical epidemiologists abstracted patient records and linked them to severe acute respiratory syndrome coronavirus 2 (SARS-CoV-2) molecular and serologic laboratory data. This population-based cohort study included NYC residents meeting the Centers for Disease Control and Prevention MIS-C case definition and admitted from March 1 to June 30, 2020; for COVID-19 hospitalizations, we included NYC residents younger than 20 years hospitalized with confirmed SARS-CoV-2 infection during the same period.^5^ Data from 163 NYC MIS-C cases were reported previously.^3^

Demographic and clinical data for individuals meeting the MIS-C criteria were described, and the incidence of MIS-C and of COVID-19 hospitalizations per 100 000 NYC residents younger than 20 years, stratified by race/ethnicity (Hispanic, non-Hispanic Black, non-Hispanic White, Asian or Pacific Islander, and multiracial or other), was calculated using 2018 population estimates based on US Census Bureau estimates. Incidence rate ratios (IRRs) and 95% CIs for MIS-C and for COVID-19 hospitalizations by race/ethnicity were calculated using SAS statistical software version 9.4 (SAS Institute).

## Results

Among 223 patients meeting the MIS-C criteria, the median (interquartile range) age was 7 (3-12) years, and 127 (57.0%) were male (Table 1). For 50 patients (22.4%) with 1 or more underlying condition, asthma (31 patients [13.9%]) and obesity (20 patients [9.0%]) were the most common conditions. SARS-CoV-2 RNA and/or antibodies were detected in 175 patients (78.5%). For 48 patients (21.5%), SARS-CoV-2 test results were negative or unavailable; these cases were included on the basis of epidemiological criteria. Race/ethnicity information was available for 184 patients (82.5%).

**Table 1.** Select Characteristics of Cases of MIS-C Among New York City Residents, March 1 to June 30, 2020

Characteristic	Patients, No. (%) (N = 223)
Age, median (interquartile range), y	7 (3-12)
Male	127 (57.0)
Underlying medical conditions^a^	
Yes	50 (22.4)
No	140 (62.8)
Unknown	33 (14.8)
Reported confirmed or suspected COVID-19–like illness before MIS-C illness onset (past 4 wk)	
Yes	19 (8.5)
No	149 (66.8)
Unknown	55 (24.7)
Reported exposure to laboratory-confirmed or suspected COVID-19 (past 4 wk)^b^	
Yes	31 (13.9)
No	171 (76.7)
Unknown	21 (9.4)
SARS-CoV-2 results, patients, No./Total (%)	
Any laboratory evidence of COVID-19	175/223 (78.5)
Positive by PCR (among cases who had a PCR test)	69/216 (31.9)
Positive by serology (among cases who had a serology test)	163/204 (79.9)
No or negative results, meets epidemiologic criteria	48/223 (21.5)

Abbreviations: COVID-19, coronavirus disease 2019; MIS-C, multisystem inflammatory syndrome in children; PCR, polymerase chain reaction; SARS-CoV-2, severe acute respiratory syndrome coronavirus 2.

^a^ Includes asthma (31 patients), obesity (20 patients), autoimmune disease (2 patients), ventricular septal defect (2 patients), cancer (1 patient), arrhythmia (1 patient), and atrial septal defect (1 patient).

^b^ Determined on the basis of the reported date of first contact.

The overall MIS-C incidence was 11.4 cases per 100 000 population younger than 20 years. Although Black children constitute 22.2% of the NYC population and 19.9% of COVID-19 hospitalizations among patients younger than 20 years, 34.4% of patients with MIS-C (75 patients) were Black (Table 2). The proportion of patients with MIS-C who were Hispanic (29.8% [65 patients]) was similar to the NYC population (35.6%), but lower than that for COVID-19 hospitalizations (40.0%). White and Asian or Pacific Islander individuals were underrepresented among MIS-C cases (28 White patients [12.8%] and 12 Asian or Pacific Islander patients [5.5%]) and COVID-19 hospitalizations (99 White patients [13.8%] and 23 Asian or Pacific Islander patients [3.2%]) compared with the NYC population (26.1% White individuals and 12.8% Asian or Pacific Islander individuals). Compared with White children, we observed a higher incidence of MIS-C among Black (IRR, 3.2; 95% CI, 2.0-4.9) and Hispanic (IRR, 1.7; 95% CI, 1.1-2.7) children and no difference among Asian or Pacific Islander children (IRR, 0.9; 95% CI, 0.4-1.7). Black (IRR, 1.7; 95% CI, 1.3-2.2) and Hispanic (IRR, 2.1; 95% CI, 1.7-2.7) children had higher COVID-19 hospitalization rates compared with White children.

**Table 2.** MIS-C Cases and COVID-19 Hospitalizations Among New York City Residents Younger Than 20 Years by Race/Ethnicity, March 1 to June 30, 2020

Race/ethnicity	MIS-C cases^a^			COVID-19 hospitalizations			NYC population, No. (%)
	No. (%)	Incidence rate^b^	IRR (95% CI)^c^	No. (%)	Incidence rate^b^	IRR (95% CI)^c^	
Asian or Pacific Islander	12 (5.5)	4.90	0.87 (0.44-1.72)	23 (3.2)	9.39	0.47 (0.30-0.75)	245 024 (12.8)
Black	75 (34.4)	17.62	3.15 (2.04-4.86)	143 (19.9)	33.59	1.70 (1.31-2.19)	425 681 (22.2)
Hispanic	65 (29.8)	9.54	1.70 (1.09-2.65)	287 (40.0)	42.11	2.13 (1.69-2.67)	681 611 (35.6)
White	28 (12.8)	5.60	1 [Reference]	99 (13.8)	19.79	1 [Reference]	500 152 (26.1)
Multiracial or other^d^	2 (0.9)	3.14	0.56 (0.13-2.36)	9 (1.3)	14.14	0.71 (0.36-1.41)	63 652 (3.3)
Missing	36 (16.5)	NA	NA	156 (21.8)	NA	NA	NA
Total	218 (100)	11.38		717 (100)	37.42		1 916 120 (100)

Abbreviations: COVID-19, coronavirus disease 2019; IRR, incidence rate ratio; MIS-C, multisystem inflammatory syndrome in children; NA, not applicable.

^a^ MIS-C cases were limited to patients aged 0 to 19 years (excluding 5 patients with MIS-C aged 20 years) to align with existing population estimates produced by New York City Department of Health and Mental Hygiene using the US Census Bureau Population Estimate Program.

^b^ Rates were calculated using number of cases as numerator, with age- and race/ethnicity-specific New York City population as denominator, per 100 000.

^c^ IRRs use the rate of White group as referent group, with 95% CI.

^d^ Other race/ethnicity includes American Indian and multiracial.

## Discussion

We present population-based data highlighting a disproportionate burden of MIS-C among Black and Hispanic children in NYC. It is unclear whether this finding represents a phenomenon distinct from the increased burden of COVID-19 in Black and Hispanic communities, because we also observed a disproportionate burden of COVID-19 hospitalizations among Black and Hispanic children. This analysis is limited by missing race/ethnicity data for most confirmed, nonhospitalized, and nonfatal COVID-19 cases in NYC, which prohibits evaluating the excess burden of MIS-C and COVID-19 hospitalizations among children of color. Furthermore, some patients meeting the MIS-C criteria may have been misclassified or not reported. Larger studies are needed to explore the relationship between MIS-C and race/ethnicity and to elucidate the impact of structural racism in perpetuating health disparities.^6^ Although MIS-C is uncommon, clinicians should be aware of the potential enhanced risk of this emerging syndrome among Black and Hispanic children.
